# 7-[(3,5-Di-*tert*-butyl-2-hy­droxy­benzyl­idene)amino]-4-methyl-2*H*-chromen-2-one

**DOI:** 10.1107/S1600536810036743

**Published:** 2010-09-18

**Authors:** Elham S. Aazam, Orhan Büyükgüngör

**Affiliations:** a Department of Chemistry, Girls Section, University of King Abdulaziz, PO Box 6171, Jeddah 21442, Saudi Arabia; b Department of Physics, Faculty of Arts and Sciences, Ondokuz Mayıs University, Kurupelit, TR-55139 Samsun, Turkey

## Abstract

The title compound, C_25_H_29_NO_3_, is a Schiff base derivative of coumarin 120. There are two structurally similar but crystallographically independent mol­ecules in the asymmetric unit. Both mol­ecules exist in *E* configurations with respect to the C=N double bonds. The dihedral angles between the coumarin and 3,5-di-*tert*-butyl-2-hy­droxy­benzyl­idene ring planes are 4.62 (7) and 14.62 (7)° for the two mol­ecules. Intra­molecular O—H⋯N hydrogen bonding involving the O—H groups and the azomethine N atoms generate *S*(6) rings. In the crystal structure, independent mol­ecules are linked by C—H⋯π inter­actions, with groups of four mol­ecules stacked along the *c* axis.

## Related literature

For the chemistry and catalytic properties of coumarin-derived Schiff base complexes, see: Youssef *et al.* (2009 ▶). For their biological and pharmacological properties, see: Kulkarni *et al.* (2009 ▶); Youssef *et al.* (2009 ▶); Ronad *et al.* (2008 ▶). For their applications as dyes and fluorescent agents, see: Kachkovski *et al.* (2004 ▶); Creaven *et al.* (2009 ▶). For related structures, see: Honda *et al.* (1996 ▶); Aazam *et al.* (2006 ▶, 2008 ▶, 2010 ▶); El Husseiny *et al.* (2008 ▶). For hydrogen-bond motifs, see: Bernstein *et al.* (1995 ▶).
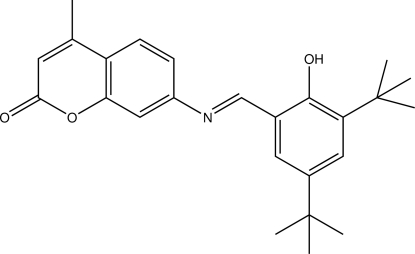


## Experimental

### 

#### Crystal data

C_25_H_29_NO_3_
*M* *_r_* = 391.49
Monoclinic, 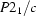

*a* = 17.6067 (14) Å
*b* = 9.6853 (5) Å
*c* = 27.237 (3) Å
β = 109.832 (6)°
*V* = 4369.2 (6) Å^3^
*Z* = 8
Mo *K*α radiation
μ = 0.08 mm^−1^
*T* = 296 K
0.32 × 0.20 × 0.05 mm

#### Data collection

Stoe IPDS 2 diffractometer
29259 measured reflections
8242 independent reflections
3118 reflections with *I* > 2σ(*I*)
*R*_int_ = 0.072

#### Refinement

*R*[*F* ^2^ > 2σ(*F* ^2^)] = 0.055
*wR*(*F* ^2^) = 0.146
*S* = 0.84
8242 reflections
524 parameters
84 restraints
H-atom parameters constrained
Δρ_max_ = 0.31 e Å^−3^
Δρ_min_ = −0.18 e Å^−3^

### 

Data collection: *X-AREA* (Stoe & Cie, 2002 ▶); cell refinement: *X-AREA*; data reduction: *X-RED* (Stoe & Cie, 2002 ▶); program(s) used to solve structure: *SHELXS97* (Sheldrick, 2008 ▶); program(s) used to refine structure: *SHELXL97* (Sheldrick, 2008 ▶); molecular graphics: *ORTEP-3 for Windows* (Farrugia, 1997 ▶); software used to prepare material for publication: *WinGX* (Farrugia, 1999 ▶).

## Supplementary Material

Crystal structure: contains datablocks I, global. DOI: 10.1107/S1600536810036743/sj5038sup1.cif

Structure factors: contains datablocks I. DOI: 10.1107/S1600536810036743/sj5038Isup2.hkl

Additional supplementary materials:  crystallographic information; 3D view; checkCIF report

## Figures and Tables

**Table 1:** Hydrogen-bond geometry (Å, °) *Cg*1 and *Cg*2 are the centroids of the C26–C31 and C1–C6 rings, respectively.

*D*—H⋯*A*	*D*—H	H⋯*A*	*D*⋯*A*	*D*—H⋯*A*
O1—H1*A*⋯N1	0.82	1.80	2.538 (4)	149
O4—H4*A*⋯N2	0.82	1.80	2.537 (4)	149
C25—H25*B*⋯*Cg*1	0.96	2.73	3.536 (4)	143
C50—H50*A*⋯*Cg*2^i^	0.96	2.80	3.571 (4)	138
C50—H50*B*⋯*Cg*1^ii^	0.96	2.91	3.569 (4)	136

Symmetry codes: (i) 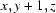; (ii) 
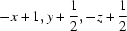
.

**Fig. 1.**
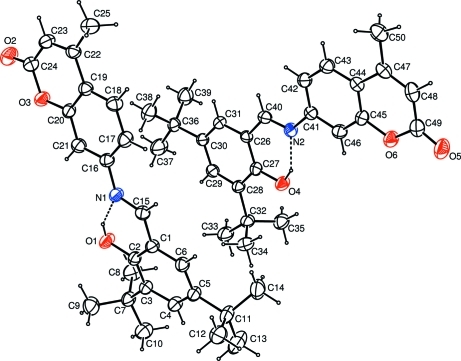
A view of molecule of molecule A and B. Displacement ellipsoids are drawn at the 30% probability level. Intramolecular hydrogen bonds are shown as dashed lines.

**Fig. 2.**
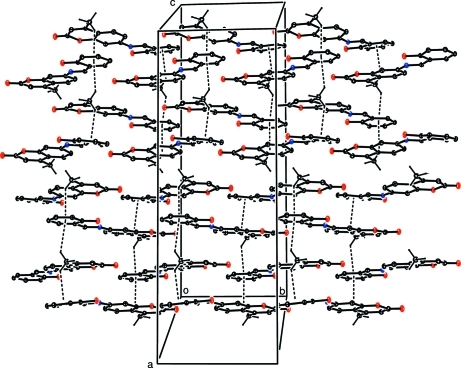
The packing diagram of the title compound, with displacement ellipsoids displayed at the 30% probability level. The t-butyl substituents on the benzene ring and H atoms not involved in C—H···π interactions (shown as dashed lines) have been omitted for clarity.

## supplementary crystallographic information

### Comment

Coumarin-derived Schiff bases are regarded as "privileged ligands" due to their ability to form complexes with different transition metals that can act as catalysts for many different reactions (Youssef *et al.*, 2009). They have been found to exhibit biological and plant regulating activities (Kulkarni *et al.*, 2009; Youssef *et al.*, 2009). They can also act as anti-inflammatory and analgesic agents (Ronad *et al.*, 2008). Several reports of their applications as dyes and fluorescent agents have appeared (Kachkovski *et al.*, 2004; Creaven *et al.*, 2009).

In a continuation of our interest in the synthesis, crystal structure elucidation (Aazam *et al.*, 2006, 2008, 2010), biological activity and photophysical properties of Schiff-base ligands incorporating a coumarin moiety and their metal complexes (El Husseiny *et al.*, 2008), we report here the crystal structure of a newly synthesized coumarin Schiff base derived from 7-amino-4-methyl coumarin and 3,5-di-t-butyl-2-hydroxybenzaldehyde.

The asymmetric unit of the title compound (Fig. 1) consists of two crystallographically independent molecules, A and B  (Honda *et al.* ,1996). The two independent molecules differ in planarity, where molecule A is more planar than molecule B, having dihedral angles between the coumarin and 3,5-di-*tert*-butyl- 2-hydroxybenzylidene ring planes of 4.64 (7)° and 14.62 (7)° for molecule A and B respectively. The planarity of both molecules is greater than that of the related 4-methyl-7-(salicylideneamino)coumarin Schiff base where the corresponding dihedral angle was 24.0 (1)° (Aazam *et al.*, 2006). The greater planarity of the title compound may explain the higher fluorescence quantum yield of 0.53 observed (compared with Φ_f_ = 0.43 for 4-methyl-7-(salicylideneamino) coumarin in DMSO).

The terminal C=O bond distances of 1.203 (4)Å and 1.209 (4)Å agree with 1.2119 (15)Å found in the related compound 4-methyl-7-(salicylideneamino) coumarin (Aazam *et al.*2006), but are shorter than that of 1.3040 (17)Å found in 8-[(1E)-1-(2-Aminophenyliminio)ethyl]-2-oxo-2*H*-chromen-7-olate (Aazam *et al.*, 2010). Intramolecular O—H···N hydrogen bonding involving the O–H groups and the  azomethine N atoms generate S(6) rings (Bernstein *et al.*, 1995). In the crystal structure, the independent molecules are linked by C—H···π interactions, with groups of four molecules stacked along the *c* axis (Fig. 2).

### Experimental

3,5-di-tertbutyl-2-hydroxybenzaldehyde (0.26 g, 1.50 mmol) in 20 ml of absolute ethanol was added to a warm solution of 7-amino-4-methyl coumarin (0.35 g, 1.5 mmol) in 30 ml of absolute ethanol. The mixture was refluxed for 3 hrs upon which a yellow solution was formed. The solvent was pumped off by rotary evaporation leaving behind an orange solid shown to be pure by NMR spectroscopy. The product was recrystallized from chloroform by slow evaporation forming orange needles. Yield (68%, 0.4 g, 1.02 mmol).

### Refinement

H atoms were positioned geometrically (C–H = 0.93 or 0.96 Å, O–H = 0.82 Å) and refined using a riding model. The *U*_iso_(H) values were set at 1.2*U*_eq_(C aromatic) and 1.5*U*_eq_(C methyl, O).

### Crystal data

**Table:**

C_25_H_29_NO_3_	*F*(000) = 1680
*M**_r_* = 391.49	*D*_x_ = 1.190 Mg m^−^^3^
Monoclinic, *P*2_1_/*c*	Mo *K*α radiation, λ = 0.71073 Å
Hall symbol: -P 2ybc	Cell parameters from 15467 reflections
*a* = 17.6067 (14) Å	θ = 1.2–26.2°
*b* = 9.6853 (5) Å	µ = 0.08 mm^−^^1^
*c* = 27.237 (3) Å	*T* = 296 K
β = 109.832 (6)°	Plate, light brown
*V* = 4369.2 (6) Å^3^	0.32 × 0.20 × 0.05 mm
*Z* = 8	

### Data collection

**Table:**

Stoe IPDS 2 diffractometer	3118 reflections with *I* > 2σ(*I*)
Radiation source: fine-focus sealed tube	*R*_int_ = 0.072
graphite	θ_max_ = 25.7°, θ_min_ = 1.2°
rotation method scans	*h* = −21→21
29259 measured reflections	*k* = −11→11
8242 independent reflections	*l* = −32→32

### Refinement

**Table:**

Refinement on *F*^2^	Primary atom site location: structure-invariant direct methods
Least-squares matrix: full	Secondary atom site location: difference Fourier map
*R*[*F*^2^ > 2σ(*F*^2^)] = 0.055	Hydrogen site location: inferred from neighbouring sites
*wR*(*F*^2^) = 0.146	H-atom parameters constrained
*S* = 0.84	*w* = 1/[σ^2^(*F*_o_^2^) + (0.0578*P*)^2^] where *P* = (*F*_o_^2^ + 2*F*_c_^2^)/3
8242 reflections	(Δ/σ)_max_ < 0.001
524 parameters	Δρ_max_ = 0.31 e Å^−^^3^
84 restraints	Δρ_min_ = −0.18 e Å^−^^3^

### Special details

**Table:**

Geometry. All e.s.d.'s (except the e.s.d. in the dihedral angle between two l.s. planes) are estimated using the full covariance matrix. The cell e.s.d.'s are taken into account individually in the estimation of e.s.d.'s in distances, angles and torsion angles; correlations between e.s.d.'s in cell parameters are only used when they are defined by crystal symmetry. An approximate (isotropic) treatment of cell e.s.d.'s is used for estimating e.s.d.'s involving l.s. planes.
Refinement. Refinement of *F*^2^ against ALL reflections. The weighted *R*-factor *wR* and goodness of fit *S* are based on *F*^2^, conventional *R*-factors *R* are based on *F*, with *F* set to zero for negative *F*^2^. The threshold expression of *F*^2^ > σ(*F*^2^) is used only for calculating *R*-factors(gt) *etc*. and is not relevant to the choice of reflections for refinement. *R*-factors based on *F*^2^ are statistically about twice as large as those based on *F*, and *R*- factors based on ALL data will be even larger.

### Fractional atomic coordinates and isotropic or equivalent isotropic displacement parameters (Å^2^)

**Table:**

	*x*	*y*	*z*	*U*_iso_*/*U*_eq_	
C1	0.71746 (19)	0.2973 (3)	0.62303 (13)	0.0465 (9)	
C2	0.7896 (2)	0.2190 (3)	0.64044 (13)	0.0472 (9)	
C3	0.8653 (2)	0.2821 (3)	0.65273 (13)	0.0479 (9)	
C4	0.8651 (2)	0.4252 (3)	0.64687 (14)	0.0555 (10)	
H4	0.9147	0.4694	0.6546	0.067*	
C5	0.7956 (2)	0.5068 (3)	0.63012 (14)	0.0503 (9)	
C6	0.7225 (2)	0.4405 (4)	0.61833 (13)	0.0503 (9)	
H6	0.6752	0.4923	0.6069	0.060*	
C7	0.9434 (2)	0.1987 (3)	0.66976 (15)	0.0552 (10)	
C8	0.9451 (2)	0.1059 (4)	0.62451 (17)	0.0831 (13)	
H8A	0.9417	0.1621	0.5948	0.100*	
H8B	0.9001	0.0435	0.6156	0.100*	
H8C	0.9945	0.0541	0.6348	0.100*	
C9	0.9506 (2)	0.1101 (4)	0.71730 (16)	0.0850 (13)	
H9A	0.9057	0.0476	0.7091	0.102*	
H9B	0.9507	0.1684	0.7458	0.102*	
H9C	1.0000	0.0584	0.7270	0.102*	
C10	1.0184 (2)	0.2909 (4)	0.68392 (17)	0.0779 (12)	
H10A	1.0193	0.3507	0.7122	0.093*	
H10B	1.0169	0.3454	0.6542	0.093*	
H10C	1.0660	0.2343	0.6943	0.093*	
C11	0.8004 (2)	0.6645 (4)	0.62607 (17)	0.0633 (11)	
C12	0.7749 (3)	0.7289 (4)	0.66932 (17)	0.0899 (14)	
H12A	0.8105	0.6982	0.7027	0.108*	
H12B	0.7206	0.7013	0.6650	0.108*	
H12C	0.7774	0.8277	0.6674	0.108*	
C13	0.8853 (3)	0.7151 (5)	0.6345 (2)	0.1178 (18)	
H13A	0.9045	0.6754	0.6086	0.141*	
H13B	0.9202	0.6880	0.6686	0.141*	
H13C	0.8851	0.8139	0.6317	0.141*	
C14	0.7431 (3)	0.7135 (4)	0.57389 (16)	0.0835 (13)	
H14A	0.6890	0.6867	0.5703	0.100*	
H14B	0.7581	0.6728	0.5463	0.100*	
H14C	0.7460	0.8123	0.5720	0.100*	
C15	0.6386 (2)	0.2320 (4)	0.60526 (13)	0.0487 (9)	
H15	0.5923	0.2859	0.5927	0.058*	
C16	0.5586 (2)	0.0272 (3)	0.58550 (13)	0.0476 (9)	
C17	0.4838 (2)	0.0864 (4)	0.55885 (14)	0.0556 (10)	
H17	0.4784	0.1818	0.5557	0.067*	
C18	0.4178 (2)	0.0014 (4)	0.53712 (14)	0.0536 (10)	
H18	0.3679	0.0415	0.5197	0.064*	
C19	0.4233 (2)	−0.1423 (4)	0.54042 (13)	0.0475 (9)	
C20	0.4982 (2)	−0.1965 (3)	0.56716 (13)	0.0484 (9)	
C21	0.5650 (2)	−0.1151 (4)	0.59003 (14)	0.0530 (9)	
H21	0.6143	−0.1556	0.6085	0.064*	
C22	0.3565 (2)	−0.2352 (4)	0.51815 (13)	0.0500 (9)	
C23	0.3719 (2)	−0.3718 (4)	0.52296 (15)	0.0629 (11)	
H23	0.3290	−0.4321	0.5085	0.075*	
C24	0.4500 (2)	−0.4303 (4)	0.54877 (14)	0.0625 (11)	
C25	0.2738 (2)	−0.1795 (4)	0.49157 (14)	0.0658 (11)	
H25A	0.2740	−0.1219	0.4629	0.079*	
H25B	0.2574	−0.1261	0.5159	0.079*	
H25C	0.2368	−0.2546	0.4787	0.079*	
N1	0.63226 (16)	0.0999 (3)	0.60680 (11)	0.0507 (8)	
O1	0.78494 (14)	0.0796 (2)	0.64396 (10)	0.0644 (7)	
H1A	0.7374	0.0565	0.6357	0.097*	
O2	0.46800 (18)	−0.5512 (3)	0.55272 (12)	0.0875 (10)	
O3	0.51209 (14)	−0.3380 (2)	0.57174 (10)	0.0606 (7)	
C26	0.67182 (18)	0.1911 (3)	0.36205 (12)	0.0424 (8)	
C27	0.7414 (2)	0.2736 (3)	0.38276 (13)	0.0479 (9)	
C28	0.81763 (19)	0.2128 (3)	0.40647 (13)	0.0478 (9)	
C29	0.8196 (2)	0.0694 (4)	0.40745 (13)	0.0520 (9)	
H29	0.8696	0.0273	0.4229	0.062*	
C30	0.7525 (2)	−0.0166 (3)	0.38701 (13)	0.0466 (9)	
C31	0.6791 (2)	0.0473 (3)	0.36445 (13)	0.0482 (9)	
H31	0.6331	−0.0066	0.3504	0.058*	
C32	0.89406 (19)	0.3001 (4)	0.43076 (15)	0.0557 (10)	
C33	0.9692 (2)	0.2112 (4)	0.45430 (18)	0.0809 (13)	
H33A	0.9765	0.1531	0.4277	0.097*	
H33B	0.9628	0.1550	0.4816	0.097*	
H33C	1.0155	0.2697	0.4684	0.097*	
C34	0.8854 (2)	0.3901 (4)	0.47456 (16)	0.0741 (12)	
H34A	0.8778	0.3323	0.5011	0.089*	
H34B	0.8396	0.4499	0.4608	0.089*	
H34C	0.9333	0.4446	0.4893	0.089*	
C35	0.9091 (2)	0.3908 (4)	0.38876 (16)	0.0744 (12)	
H35A	0.8636	0.4506	0.3737	0.089*	
H35B	0.9163	0.3332	0.3620	0.089*	
H35C	0.9567	0.4455	0.4043	0.089*	
C36	0.7602 (2)	−0.1747 (4)	0.39020 (15)	0.0548 (10)	
C37	0.8450 (3)	−0.2233 (4)	0.4182 (2)	0.1033 (16)	
H37A	0.8633	−0.1883	0.4532	0.124*	
H37B	0.8798	−0.1904	0.4002	0.124*	
H37C	0.8460	−0.3224	0.4190	0.124*	
C38	0.7046 (3)	−0.2350 (4)	0.41652 (17)	0.0836 (13)	
H38A	0.6499	−0.2097	0.3972	0.100*	
H38B	0.7193	−0.1998	0.4515	0.100*	
H38C	0.7095	−0.3338	0.4176	0.100*	
C39	0.7345 (3)	−0.2358 (4)	0.33471 (16)	0.0851 (13)	
H39A	0.7690	−0.2006	0.3169	0.102*	
H39B	0.6796	−0.2103	0.3159	0.102*	
H39C	0.7388	−0.3346	0.3368	0.102*	
C40	0.59273 (19)	0.2538 (4)	0.33832 (13)	0.0492 (9)	
H40	0.5476	0.1978	0.3246	0.059*	
C41	0.5103 (2)	0.4573 (3)	0.31500 (13)	0.0483 (9)	
C42	0.4340 (2)	0.3969 (4)	0.29606 (14)	0.0549 (10)	
H42	0.4285	0.3014	0.2958	0.066*	
C43	0.3672 (2)	0.4796 (4)	0.27784 (13)	0.0550 (10)	
H43	0.3166	0.4380	0.2653	0.066*	
C44	0.3715 (2)	0.6238 (4)	0.27722 (13)	0.0467 (9)	
C45	0.4483 (2)	0.6801 (3)	0.29646 (14)	0.0495 (9)	
C46	0.5164 (2)	0.5999 (4)	0.31460 (14)	0.0542 (10)	
H46	0.5670	0.6415	0.3267	0.065*	
C47	0.3032 (2)	0.7170 (4)	0.25793 (13)	0.0527 (9)	
C48	0.3177 (3)	0.8536 (4)	0.26011 (15)	0.0642 (11)	
H48	0.2738	0.9128	0.2475	0.077*	
C49	0.3967 (3)	0.9138 (4)	0.28057 (15)	0.0608 (10)	
C50	0.2196 (2)	0.6579 (4)	0.23672 (15)	0.0687 (11)	
H50A	0.2094	0.6025	0.2631	0.082*	
H50B	0.2150	0.6016	0.2068	0.082*	
H50C	0.1810	0.7316	0.2267	0.082*	
N2	0.58451 (16)	0.3866 (3)	0.33607 (11)	0.0516 (8)	
O4	0.73564 (13)	0.4132 (2)	0.38099 (9)	0.0616 (7)	
H4A	0.6884	0.4357	0.3664	0.092*	
O5	0.41309 (18)	1.0345 (3)	0.28431 (11)	0.0795 (9)	
O6	0.46070 (14)	0.8222 (2)	0.29799 (10)	0.0615 (7)	

### Atomic displacement parameters (Å^2^)

**Table:**

	*U*^11^	*U*^22^	*U*^33^	*U*^12^	*U*^13^	*U*^23^
C1	0.046 (2)	0.045 (2)	0.048 (2)	−0.0058 (17)	0.0157 (16)	−0.0017 (17)
C2	0.051 (2)	0.037 (2)	0.053 (2)	−0.0006 (16)	0.0169 (18)	0.0018 (16)
C3	0.050 (2)	0.041 (2)	0.054 (2)	−0.0001 (16)	0.0187 (17)	0.0008 (17)
C4	0.052 (2)	0.046 (2)	0.068 (3)	−0.0034 (18)	0.0206 (19)	0.0006 (19)
C5	0.048 (2)	0.040 (2)	0.061 (2)	−0.0013 (17)	0.0180 (18)	0.0041 (18)
C6	0.048 (2)	0.047 (2)	0.055 (2)	0.0013 (17)	0.0165 (17)	0.0039 (18)
C7	0.049 (2)	0.044 (2)	0.070 (3)	0.0044 (17)	0.0160 (18)	0.004 (2)
C8	0.073 (3)	0.071 (2)	0.105 (3)	0.009 (2)	0.031 (2)	−0.011 (2)
C9	0.069 (2)	0.082 (3)	0.094 (3)	0.007 (2)	0.014 (2)	0.022 (2)
C10	0.056 (2)	0.068 (2)	0.103 (3)	0.002 (2)	0.018 (2)	0.000 (2)
C11	0.057 (2)	0.041 (2)	0.093 (3)	0.0015 (18)	0.027 (2)	0.015 (2)
C12	0.109 (3)	0.052 (2)	0.091 (3)	0.004 (2)	0.011 (2)	0.000 (2)
C13	0.090 (3)	0.068 (3)	0.189 (4)	−0.011 (2)	0.039 (3)	0.030 (3)
C14	0.106 (3)	0.059 (2)	0.085 (3)	0.008 (2)	0.032 (2)	0.018 (2)
C15	0.044 (2)	0.051 (2)	0.050 (2)	−0.0001 (17)	0.0161 (17)	−0.0006 (18)
C16	0.041 (2)	0.050 (2)	0.051 (2)	−0.0034 (17)	0.0148 (16)	−0.0006 (18)
C17	0.057 (2)	0.044 (2)	0.067 (3)	0.0048 (18)	0.0220 (19)	0.0015 (19)
C18	0.041 (2)	0.057 (2)	0.061 (2)	0.0024 (18)	0.0146 (18)	−0.0032 (19)
C19	0.045 (2)	0.051 (2)	0.047 (2)	−0.0041 (17)	0.0162 (17)	−0.0020 (17)
C20	0.053 (2)	0.042 (2)	0.051 (2)	−0.0045 (18)	0.0192 (18)	0.0023 (18)
C21	0.045 (2)	0.054 (2)	0.056 (2)	0.0016 (18)	0.0116 (17)	0.0024 (19)
C22	0.049 (2)	0.060 (2)	0.042 (2)	−0.0094 (18)	0.0159 (17)	−0.0056 (18)
C23	0.066 (3)	0.060 (3)	0.060 (3)	−0.023 (2)	0.018 (2)	−0.008 (2)
C24	0.071 (3)	0.053 (3)	0.058 (3)	−0.012 (2)	0.015 (2)	−0.003 (2)
C25	0.058 (2)	0.078 (2)	0.062 (2)	−0.010 (2)	0.0209 (18)	−0.012 (2)
N1	0.0504 (18)	0.0427 (18)	0.058 (2)	−0.0060 (14)	0.0171 (14)	−0.0006 (15)
O1	0.0574 (16)	0.0368 (14)	0.091 (2)	−0.0038 (12)	0.0151 (14)	0.0042 (13)
O2	0.104 (2)	0.0444 (17)	0.106 (3)	−0.0065 (16)	0.0258 (19)	0.0004 (16)
O3	0.0566 (16)	0.0443 (14)	0.0735 (18)	−0.0036 (12)	0.0125 (13)	0.0014 (13)
C26	0.0369 (19)	0.047 (2)	0.041 (2)	−0.0025 (16)	0.0111 (15)	0.0019 (16)
C27	0.053 (2)	0.039 (2)	0.050 (2)	0.0032 (17)	0.0146 (18)	0.0027 (17)
C28	0.0397 (19)	0.046 (2)	0.055 (2)	−0.0021 (16)	0.0128 (16)	0.0017 (17)
C29	0.044 (2)	0.050 (2)	0.056 (2)	0.0050 (17)	0.0088 (17)	0.0042 (18)
C30	0.044 (2)	0.0399 (19)	0.051 (2)	0.0024 (16)	0.0105 (16)	0.0009 (17)
C31	0.051 (2)	0.042 (2)	0.050 (2)	−0.0052 (17)	0.0156 (17)	−0.0020 (17)
C32	0.039 (2)	0.053 (2)	0.069 (3)	−0.0025 (17)	0.0116 (18)	0.002 (2)
C33	0.055 (2)	0.069 (2)	0.104 (3)	−0.007 (2)	0.007 (2)	0.005 (2)
C34	0.063 (2)	0.071 (2)	0.081 (3)	−0.021 (2)	0.016 (2)	−0.010 (2)
C35	0.064 (2)	0.073 (2)	0.089 (3)	−0.012 (2)	0.029 (2)	0.004 (2)
C36	0.053 (2)	0.046 (2)	0.062 (3)	0.0054 (18)	0.0156 (19)	0.0056 (19)
C37	0.087 (3)	0.058 (2)	0.140 (4)	0.006 (2)	0.006 (3)	0.009 (2)
C38	0.103 (3)	0.057 (2)	0.096 (3)	−0.003 (2)	0.042 (2)	0.004 (2)
C39	0.109 (3)	0.065 (2)	0.085 (3)	0.003 (2)	0.037 (2)	−0.001 (2)
C40	0.044 (2)	0.054 (2)	0.047 (2)	−0.0025 (17)	0.0110 (16)	−0.0019 (18)
C41	0.046 (2)	0.047 (2)	0.048 (2)	0.0016 (17)	0.0110 (17)	0.0017 (17)
C42	0.053 (2)	0.046 (2)	0.061 (2)	0.0044 (18)	0.0130 (18)	0.0003 (18)
C43	0.049 (2)	0.055 (2)	0.058 (3)	0.0004 (19)	0.0145 (19)	0.0001 (19)
C44	0.046 (2)	0.051 (2)	0.042 (2)	0.0050 (17)	0.0138 (17)	0.0016 (17)
C45	0.055 (2)	0.040 (2)	0.055 (2)	0.0025 (18)	0.0209 (18)	0.0015 (17)
C46	0.041 (2)	0.057 (2)	0.062 (3)	−0.0017 (18)	0.0142 (18)	0.002 (2)
C47	0.049 (2)	0.067 (3)	0.042 (2)	0.0101 (19)	0.0150 (17)	0.0022 (19)
C48	0.074 (3)	0.062 (3)	0.060 (3)	0.023 (2)	0.027 (2)	0.014 (2)
C49	0.074 (3)	0.054 (2)	0.057 (3)	0.009 (2)	0.025 (2)	0.006 (2)
C50	0.057 (2)	0.078 (2)	0.067 (2)	0.017 (2)	0.0163 (19)	0.002 (2)
N2	0.0433 (17)	0.0456 (18)	0.061 (2)	0.0070 (14)	0.0115 (14)	0.0054 (15)
O4	0.0467 (15)	0.0432 (15)	0.0863 (19)	0.0006 (12)	0.0115 (13)	0.0039 (13)
O5	0.104 (2)	0.0458 (17)	0.090 (2)	0.0101 (15)	0.0349 (18)	0.0071 (15)
O6	0.0628 (17)	0.0441 (15)	0.0746 (19)	0.0052 (13)	0.0194 (14)	0.0014 (13)

### Geometric parameters (Å, °)

**Table:**

C1—C6	1.398 (5)	C26—C31	1.398 (5)
C1—C2	1.417 (4)	C26—C27	1.410 (4)
C1—C15	1.452 (4)	C26—C40	1.454 (4)
C2—O1	1.358 (4)	C27—O4	1.356 (4)
C2—C3	1.399 (4)	C27—C28	1.406 (4)
C3—C4	1.395 (5)	C28—C29	1.390 (5)
C3—C7	1.526 (4)	C28—C32	1.536 (5)
C4—C5	1.397 (4)	C29—C30	1.398 (4)
C4—H4	0.9300	C29—H29	0.9300
C5—C6	1.375 (4)	C30—C31	1.375 (4)
C5—C11	1.536 (5)	C30—C36	1.537 (5)
C6—H6	0.9300	C31—H31	0.9300
C7—C9	1.522 (5)	C32—C33	1.525 (5)
C7—C10	1.531 (5)	C32—C34	1.526 (5)
C7—C8	1.533 (5)	C32—C35	1.535 (5)
C8—H8A	0.9600	C33—H33A	0.9600
C8—H8B	0.9600	C33—H33B	0.9600
C8—H8C	0.9600	C33—H33C	0.9600
C9—H9A	0.9600	C34—H34A	0.9600
C9—H9B	0.9600	C34—H34B	0.9600
C9—H9C	0.9600	C34—H34C	0.9600
C10—H10A	0.9600	C35—H35A	0.9600
C10—H10B	0.9600	C35—H35B	0.9600
C10—H10C	0.9600	C35—H35C	0.9600
C11—C14	1.513 (5)	C36—C37	1.503 (5)
C11—C13	1.515 (5)	C36—C38	1.513 (5)
C11—C12	1.529 (6)	C36—C39	1.541 (5)
C12—H12A	0.9600	C37—H37A	0.9600
C12—H12B	0.9600	C37—H37B	0.9600
C12—H12C	0.9600	C37—H37C	0.9600
C13—H13A	0.9600	C38—H38A	0.9600
C13—H13B	0.9600	C38—H38B	0.9600
C13—H13C	0.9600	C38—H38C	0.9600
C14—H14A	0.9600	C39—H39A	0.9600
C14—H14B	0.9600	C39—H39B	0.9600
C14—H14C	0.9600	C39—H39C	0.9600
C15—N1	1.286 (4)	C40—N2	1.294 (4)
C15—H15	0.9300	C40—H40	0.9300
C16—C21	1.384 (5)	C41—C46	1.386 (5)
C16—C17	1.393 (4)	C41—C42	1.394 (5)
C16—N1	1.416 (4)	C41—N2	1.413 (4)
C17—C18	1.382 (5)	C42—C43	1.369 (4)
C17—H17	0.9300	C42—H42	0.9300
C18—C19	1.396 (5)	C43—C44	1.399 (5)
C18—H18	0.9300	C43—H43	0.9300
C19—C20	1.376 (5)	C44—C45	1.386 (5)
C19—C22	1.442 (5)	C44—C47	1.453 (4)
C20—C21	1.378 (4)	C45—C46	1.372 (4)
C20—O3	1.390 (4)	C45—O6	1.392 (4)
C21—H21	0.9300	C46—H46	0.9300
C22—C23	1.348 (5)	C47—C48	1.344 (5)
C22—C25	1.489 (5)	C47—C50	1.500 (5)
C23—C24	1.432 (5)	C48—C49	1.434 (5)
C23—H23	0.9300	C48—H48	0.9300
C24—O2	1.208 (4)	C49—O5	1.201 (4)
C24—O3	1.386 (4)	C49—O6	1.385 (4)
C25—H25A	0.9600	C50—H50A	0.9600
C25—H25B	0.9600	C50—H50B	0.9600
C25—H25C	0.9600	C50—H50C	0.9600
O1—H1A	0.8200	O4—H4A	0.8200
			
C6—C1—C2	119.0 (3)	C31—C26—C27	119.5 (3)
C6—C1—C15	119.0 (3)	C31—C26—C40	119.7 (3)
C2—C1—C15	121.7 (3)	C27—C26—C40	120.8 (3)
O1—C2—C3	119.6 (3)	O4—C27—C28	118.8 (3)
O1—C2—C1	119.1 (3)	O4—C27—C26	120.5 (3)
C3—C2—C1	121.3 (3)	C28—C27—C26	120.7 (3)
C4—C3—C2	116.2 (3)	C29—C28—C27	116.2 (3)
C4—C3—C7	121.8 (3)	C29—C28—C32	121.9 (3)
C2—C3—C7	121.9 (3)	C27—C28—C32	121.9 (3)
C3—C4—C5	124.5 (3)	C28—C29—C30	125.1 (3)
C3—C4—H4	117.7	C28—C29—H29	117.4
C5—C4—H4	117.7	C30—C29—H29	117.4
C6—C5—C4	117.4 (3)	C31—C30—C29	116.7 (3)
C6—C5—C11	121.2 (3)	C31—C30—C36	121.7 (3)
C4—C5—C11	121.5 (3)	C29—C30—C36	121.6 (3)
C5—C6—C1	121.6 (3)	C30—C31—C26	121.8 (3)
C5—C6—H6	119.2	C30—C31—H31	119.1
C1—C6—H6	119.2	C26—C31—H31	119.1
C9—C7—C3	111.3 (3)	C33—C32—C34	107.2 (3)
C9—C7—C10	107.4 (3)	C33—C32—C35	107.0 (3)
C3—C7—C10	112.3 (3)	C34—C32—C35	110.1 (3)
C9—C7—C8	109.6 (3)	C33—C32—C28	112.2 (3)
C3—C7—C8	109.1 (3)	C34—C32—C28	110.2 (3)
C10—C7—C8	107.1 (3)	C35—C32—C28	110.0 (3)
C7—C8—H8A	109.5	C32—C33—H33A	109.5
C7—C8—H8B	109.5	C32—C33—H33B	109.5
H8A—C8—H8B	109.5	H33A—C33—H33B	109.5
C7—C8—H8C	109.5	C32—C33—H33C	109.5
H8A—C8—H8C	109.5	H33A—C33—H33C	109.5
H8B—C8—H8C	109.5	H33B—C33—H33C	109.5
C7—C9—H9A	109.5	C32—C34—H34A	109.5
C7—C9—H9B	109.5	C32—C34—H34B	109.5
H9A—C9—H9B	109.5	H34A—C34—H34B	109.5
C7—C9—H9C	109.5	C32—C34—H34C	109.5
H9A—C9—H9C	109.5	H34A—C34—H34C	109.5
H9B—C9—H9C	109.5	H34B—C34—H34C	109.5
C7—C10—H10A	109.5	C32—C35—H35A	109.5
C7—C10—H10B	109.5	C32—C35—H35B	109.5
H10A—C10—H10B	109.5	H35A—C35—H35B	109.5
C7—C10—H10C	109.5	C32—C35—H35C	109.5
H10A—C10—H10C	109.5	H35A—C35—H35C	109.5
H10B—C10—H10C	109.5	H35B—C35—H35C	109.5
C14—C11—C13	110.5 (4)	C37—C36—C38	109.2 (3)
C14—C11—C12	108.6 (3)	C37—C36—C30	113.2 (3)
C13—C11—C12	106.5 (4)	C38—C36—C30	110.6 (3)
C14—C11—C5	110.1 (3)	C37—C36—C39	106.7 (3)
C13—C11—C5	112.8 (3)	C38—C36—C39	107.1 (3)
C12—C11—C5	108.2 (3)	C30—C36—C39	109.7 (3)
C11—C12—H12A	109.5	C36—C37—H37A	109.5
C11—C12—H12B	109.5	C36—C37—H37B	109.5
H12A—C12—H12B	109.5	H37A—C37—H37B	109.5
C11—C12—H12C	109.5	C36—C37—H37C	109.5
H12A—C12—H12C	109.5	H37A—C37—H37C	109.5
H12B—C12—H12C	109.5	H37B—C37—H37C	109.5
C11—C13—H13A	109.5	C36—C38—H38A	109.5
C11—C13—H13B	109.5	C36—C38—H38B	109.5
H13A—C13—H13B	109.5	H38A—C38—H38B	109.5
C11—C13—H13C	109.5	C36—C38—H38C	109.5
H13A—C13—H13C	109.5	H38A—C38—H38C	109.5
H13B—C13—H13C	109.5	H38B—C38—H38C	109.5
C11—C14—H14A	109.5	C36—C39—H39A	109.5
C11—C14—H14B	109.5	C36—C39—H39B	109.5
H14A—C14—H14B	109.5	H39A—C39—H39B	109.5
C11—C14—H14C	109.5	C36—C39—H39C	109.5
H14A—C14—H14C	109.5	H39A—C39—H39C	109.5
H14B—C14—H14C	109.5	H39B—C39—H39C	109.5
N1—C15—C1	120.4 (3)	N2—C40—C26	120.7 (3)
N1—C15—H15	119.8	N2—C40—H40	119.6
C1—C15—H15	119.8	C26—C40—H40	119.6
C21—C16—C17	119.3 (3)	C46—C41—C42	118.9 (3)
C21—C16—N1	115.0 (3)	C46—C41—N2	115.0 (3)
C17—C16—N1	125.6 (3)	C42—C41—N2	126.1 (3)
C18—C17—C16	119.1 (3)	C43—C42—C41	119.4 (4)
C18—C17—H17	120.4	C43—C42—H42	120.3
C16—C17—H17	120.4	C41—C42—H42	120.3
C17—C18—C19	122.4 (3)	C42—C43—C44	123.0 (4)
C17—C18—H18	118.8	C42—C43—H43	118.5
C19—C18—H18	118.8	C44—C43—H43	118.5
C20—C19—C18	116.6 (3)	C45—C44—C43	116.0 (3)
C20—C19—C22	118.9 (3)	C45—C44—C47	118.3 (3)
C18—C19—C22	124.5 (3)	C43—C44—C47	125.7 (3)
C19—C20—C21	122.6 (3)	C46—C45—C44	122.3 (3)
C19—C20—O3	122.1 (3)	C46—C45—O6	116.1 (3)
C21—C20—O3	115.2 (3)	C44—C45—O6	121.6 (3)
C20—C21—C16	119.9 (3)	C45—C46—C41	120.4 (3)
C20—C21—H21	120.1	C45—C46—H46	119.8
C16—C21—H21	120.1	C41—C46—H46	119.8
C23—C22—C19	117.6 (3)	C48—C47—C44	118.3 (4)
C23—C22—C25	122.3 (3)	C48—C47—C50	122.7 (3)
C19—C22—C25	120.1 (3)	C44—C47—C50	119.0 (3)
C22—C23—C24	124.4 (4)	C47—C48—C49	124.2 (4)
C22—C23—H23	117.8	C47—C48—H48	117.9
C24—C23—H23	117.8	C49—C48—H48	117.9
O2—C24—O3	116.2 (4)	O5—C49—O6	116.7 (4)
O2—C24—C23	127.4 (4)	O5—C49—C48	127.1 (4)
O3—C24—C23	116.4 (4)	O6—C49—C48	116.2 (3)
C22—C25—H25A	109.5	C47—C50—H50A	109.5
C22—C25—H25B	109.5	C47—C50—H50B	109.5
H25A—C25—H25B	109.5	H50A—C50—H50B	109.5
C22—C25—H25C	109.5	C47—C50—H50C	109.5
H25A—C25—H25C	109.5	H50A—C50—H50C	109.5
H25B—C25—H25C	109.5	H50B—C50—H50C	109.5
C15—N1—C16	124.0 (3)	C40—N2—C41	125.0 (3)
C2—O1—H1A	109.5	C27—O4—H4A	109.5
C24—O3—C20	120.5 (3)	C49—O6—C45	121.4 (3)
			
C6—C1—C2—O1	−178.6 (3)	C31—C26—C27—O4	−179.8 (3)
C15—C1—C2—O1	−4.6 (5)	C40—C26—C27—O4	0.2 (5)
C6—C1—C2—C3	−0.2 (5)	C31—C26—C27—C28	−1.0 (5)
C15—C1—C2—C3	173.8 (3)	C40—C26—C27—C28	178.9 (3)
O1—C2—C3—C4	178.4 (3)	O4—C27—C28—C29	179.5 (3)
C1—C2—C3—C4	0.0 (5)	C26—C27—C28—C29	0.7 (5)
O1—C2—C3—C7	0.4 (5)	O4—C27—C28—C32	0.6 (5)
C1—C2—C3—C7	−177.9 (3)	C26—C27—C28—C32	−178.2 (3)
C2—C3—C4—C5	0.3 (5)	C27—C28—C29—C30	−0.1 (5)
C7—C3—C4—C5	178.3 (3)	C32—C28—C29—C30	178.8 (3)
C3—C4—C5—C6	−0.5 (6)	C28—C29—C30—C31	−0.2 (5)
C3—C4—C5—C11	178.3 (4)	C28—C29—C30—C36	−179.5 (4)
C4—C5—C6—C1	0.3 (5)	C29—C30—C31—C26	−0.1 (5)
C11—C5—C6—C1	−178.5 (4)	C36—C30—C31—C26	179.2 (4)
C2—C1—C6—C5	0.1 (5)	C27—C26—C31—C30	0.7 (5)
C15—C1—C6—C5	−174.1 (3)	C40—C26—C31—C30	−179.3 (3)
C4—C3—C7—C9	125.7 (4)	C29—C28—C32—C33	0.8 (5)
C2—C3—C7—C9	−56.5 (5)	C27—C28—C32—C33	179.7 (3)
C4—C3—C7—C10	5.3 (5)	C29—C28—C32—C34	−118.6 (4)
C2—C3—C7—C10	−176.9 (3)	C27—C28—C32—C34	60.3 (5)
C4—C3—C7—C8	−113.3 (4)	C29—C28—C32—C35	119.8 (4)
C2—C3—C7—C8	64.6 (4)	C27—C28—C32—C35	−61.3 (4)
C6—C5—C11—C14	−49.3 (5)	C31—C30—C36—C37	−177.7 (4)
C4—C5—C11—C14	132.0 (4)	C29—C30—C36—C37	1.5 (5)
C6—C5—C11—C13	−173.3 (4)	C31—C30—C36—C38	−54.7 (5)
C4—C5—C11—C13	8.0 (6)	C29—C30—C36—C38	124.6 (4)
C6—C5—C11—C12	69.2 (5)	C31—C30—C36—C39	63.2 (5)
C4—C5—C11—C12	−109.5 (4)	C29—C30—C36—C39	−117.5 (4)
C6—C1—C15—N1	176.7 (3)	C31—C26—C40—N2	179.4 (4)
C2—C1—C15—N1	2.7 (5)	C27—C26—C40—N2	−0.5 (5)
C21—C16—C17—C18	−0.6 (5)	C46—C41—C42—C43	−0.3 (5)
N1—C16—C17—C18	175.7 (3)	N2—C41—C42—C43	178.5 (3)
C16—C17—C18—C19	−0.6 (6)	C41—C42—C43—C44	0.0 (6)
C17—C18—C19—C20	0.8 (6)	C42—C43—C44—C45	−0.1 (5)
C17—C18—C19—C22	−179.6 (3)	C42—C43—C44—C47	179.3 (3)
C18—C19—C20—C21	0.3 (5)	C43—C44—C45—C46	0.7 (5)
C22—C19—C20—C21	−179.4 (3)	C47—C44—C45—C46	−178.8 (3)
C18—C19—C20—O3	−177.9 (3)	C43—C44—C45—O6	−180.0 (3)
C22—C19—C20—O3	2.5 (5)	C47—C44—C45—O6	0.6 (5)
C19—C20—C21—C16	−1.5 (6)	C44—C45—C46—C41	−1.0 (6)
O3—C20—C21—C16	176.8 (3)	O6—C45—C46—C41	179.6 (3)
C17—C16—C21—C20	1.6 (5)	C42—C41—C46—C45	0.8 (5)
N1—C16—C21—C20	−175.0 (3)	N2—C41—C46—C45	−178.1 (3)
C20—C19—C22—C23	−2.7 (5)	C45—C44—C47—C48	−0.6 (5)
C18—C19—C22—C23	177.6 (4)	C43—C44—C47—C48	−180.0 (4)
C20—C19—C22—C25	176.6 (3)	C45—C44—C47—C50	179.7 (3)
C18—C19—C22—C25	−3.1 (5)	C43—C44—C47—C50	0.3 (5)
C19—C22—C23—C24	0.2 (6)	C44—C47—C48—C49	−0.3 (6)
C25—C22—C23—C24	−179.1 (3)	C50—C47—C48—C49	179.4 (3)
C22—C23—C24—O2	−177.2 (4)	C47—C48—C49—O5	−178.6 (4)
C22—C23—C24—O3	2.6 (6)	C47—C48—C49—O6	1.2 (6)
C1—C15—N1—C16	−173.4 (3)	C26—C40—N2—C41	−178.4 (3)
C21—C16—N1—C15	178.4 (4)	C46—C41—N2—C40	−177.2 (4)
C17—C16—N1—C15	2.0 (6)	C42—C41—N2—C40	3.9 (6)
O2—C24—O3—C20	176.9 (4)	O5—C49—O6—C45	178.6 (4)
C23—C24—O3—C20	−2.9 (5)	C48—C49—O6—C45	−1.2 (5)
C19—C20—O3—C24	0.5 (5)	C46—C45—O6—C49	179.8 (3)
C21—C20—O3—C24	−177.8 (3)	C44—C45—O6—C49	0.4 (5)

### Hydrogen-bond geometry (Å, °)

**Table:**

Cg1 and Cg2 are the centroids of the C26–C31 and C1–C6 rings, respectively.

**Table:**

*D*—H···*A*	*D*—H	H···*A*	*D*···*A*	*D*—H···*A*
O1—H1A···N1	0.82	1.80	2.538 (4)	149
O4—H4A···N2	0.82	1.80	2.537 (4)	149
C25—H25B···Cg1	0.96	2.73	3.536 (4)	143
C50—H50A···Cg2^i^	0.96	2.80	3.571 (4)	138
C50—H50B···Cg1^ii^	0.96	2.91	3.569 (4)	136

Symmetry codes: (i) *x*, *y*+1, *z*; (ii) −*x*+1, *y*+1/2, −*z*+1/2.
